# Online Visual Detection System for Head Warping and Lower Buckling of Hot-Rolled Rough Slab

**DOI:** 10.3390/s25061662

**Published:** 2025-03-07

**Authors:** Shitao Ge, Yan Peng, Jianliang Sun, Licheng Han

**Affiliations:** 1 National Engineering Research Center for Equipment and Technology of Cold Strip Rolling, Yanshan University, Qinhuangdao 066004, China; geshitaoysu@stumail.ysu.edu.cn (S.G.); sunjianliang@ysu.edu.cn (J.S.); hanlicheng@stumail.ysu.edu.cn (L.H.); 2 State Key Laboratory of Crane Technology, Yanshan University, Qinhuangdao 066004, China

**Keywords:** rough-rolled slab, visual inspection, cascaded filter, Canny’s algorithm, online real-time detection

## Abstract

The real-time measurement of head warping and lower buckling during the production process of rough-rolled slabs has long been a persistent technical problem at the production site. Currently, the detection of head warping and lower buckling in the production site relies on workers’ operational experience for manual observation or measurement during machine downtime. In this paper, an online real-time detection system for the head warping and lower buckling of rough-rolled slab in hot continuous rolling based on visual detection is proposed, and a cascade filter based on morphological processing is developed, which can effectively remove the noise in the field environment and smooth the edge profile of the slab. A precise measurement and analysis method based on points and lines is proposed, which determines the precise values by subtracting the distance from the corner-point at the top of slab to the straight line at its lower edge from that between its upper and lower edges. The detection system in industrial applications has demonstrated high accuracy: detection error ≤ ±5 mm, type recognition rate ≥ 99%. Meeting on-site industrial production requirements.

## 1. Introduction

During the hot continuous rough rolling process, the most significant shape defects are the warping and lower buckling of the head of the slab [1,2,3,4]. When severe warping occurs, it not only affects the quality of the slab but also impacts the working rolls and water cooling devices, reducing their service life. Additionally, it can affect the control of rolling stability in subsequent finishing rolling processes. When the slab is severely warped, it may even cause major production accidents, such as scrap steel and stacked steel [5]. When the slab is severely lower buckled, it will collide with the frame rollers and roller tracks, causing many disadvantages in production. Currently, there are few accurate detection methods for head warping and lower buckling in rough-rolled slabs. Most units mainly rely on manual monitoring and adjusting the speed difference between rolls to improve the situation of slab head warping and lower buckling. Due to low accuracy and poor stability in manual judgment, it is difficult to accurately characterize and evaluate the situation of slab head warping and lower buckling, resulting in poor control effect in industrial production sites. Here are some ready-made solutions for industrial applications: GEOMETRIX-PL of NK NORDINKRAFT, Schaulnslandstr, 1675196 Remchingen and country from Germany, which is available for non-contact measurement of length, flatness, width, and camber angles for sheets, strips, and slabs. LJ-X8000 2D/3D of KEYENCE, Osaka and country from Japan is suitable for slab size measurement and appearance inspection. ZLDS210 of ZSY Group Ltd., London and country from UK, which can measure the profile of large steel plates and pipes. The above-mentioned ready-made solutions for industrial applications have basically realized online real-time detection of the plate, and they basically determine the method of line laser measurement for the real-time detection of the slab, while the detection system described in this paper uses the way of the area array industrial camera for the real-time detection of the slab, and it only needs to shoot the slab head image to complete the quantitative calculation of the slab contour and head bending. Compared to the above products, our method will be simpler, have a faster response and a lower cost. In recent years, with the development of machine vision technology, conditions have been created for the online detection of slab head warping and lower buckling as well as unmanned automatic steel rolling. 

Extensive research has been conducted by domestic and foreign scholars to address the issues of head warping and lower buckling in visual inspection of rough-rolled slabs. Xu [6] proposed a (MSF-Net) based on (DMF) extractor, which proposes the diversity of feature receptive fields while reducing the amount of calculation; the feature maps of the middle layer with different sizes of receptive fields are merged to increase the richness of the receptive fields of the last layer of feature maps; the residual shortcut connections, batch normalization layer, and average pooling layer are used to replace the fully connected layer to improve training efficiency, and make the multi-scale feature learning ability more balanced at the same time. Mei Xi [7] set up a head warping detection system using a CCD camera, allowing operators to make on-site adjustments. On the basis of studying the stripe projection measurement technology, Li [8] proposed a divergent multi-line laser projection 3D simulation system, which can simulate a divergent multi-line laser projection 3D measurement system in a computer environment. Yao and Liu [9] designed a telecentric surface reconstruction system for surface inspection, which utilizes a monocular camera with telecentric lens, a line laser generator, and a one-dimensional displacement platform to accurately reconstruct the surface. ME Mamoun [10] described a hybrid method based on combining the most efficient classification techniques. A trained convolutional neural network (CNN) was applied to extract features from character images. Then, a support vector machine (SVM) was used for classification. By combining CNN and SVM, the aim is to exploit both technologies’ strengths. Four hybrid models are proposed in this work. Several databases such as HACDB, HIJJA, AHCD, and MNIST were used to evaluate them. The results obtained are satisfactory compared to similar studies in the literature, with a test accuracy of 89.7, 88.8, 97.3, and 99.4%, respectively. Bouguettaya et al. [11] proposed a deep ensemble transfer learning-based method to classify hot-rolled steel strip surface defects by combining MobileNet-V2 and Xception architectures, achieving good results. They were able to achieve an accuracy of 99.72%, outperforming the one branch MobileNet-V2 and Xception models, which achieved 98.61% and 99.17%. Boudiaf A [12] proposed using a pre-trained AlexNet model combined with an SVM classifier, achieving an accuracy of 99.7%. They investigated the impact of layer selection on classification accuracy. Lu [13] proposed an accurate and easy-to-implement method for online non-destructive full-field three-dimensional displacement measurement using electronic speckle pattern interferometry based on a single-illumination detection path. Their work provides important references for future research and applications. Pang W [14] proposed an efficient defect detector called Vision Grapher with Hadamard, which employs a novel attention mechanism (HDmA) to establish local-to-local relationships within an image and integrates global relationships via graph convolution. With the HDmA module, we can not only fuse information under the same field of view, but also under different fields of view, which significantly enhances the richness of the acquired features. Chao Zhao [15] proposed a model, named RDD-YOLO, based on YOLOv5 for steel surface defect detection. Firstly, the backbone component is consisted of Res2Net blocks to enlarge the receptive field and extract features of various scales. Feng [16] presented a date augmentation method named SDDA to augment the surface defects of cold rolling mill steel. Yuansong Wang [17] presented a convolutional neural network (CNN) based image semantic segmentation technique for pixel-level classification of DIC strain field images. Shuzong Yan [18] proposed a non-contact strip deviation online measurement scheme based on machine vision technology and image detection. Shuzong Yan and Dong Xu [19] proposed a measurement system based on line-structured light vision, however, the detection speed is relatively slow due to the use of structured light. They have provided important references for future research and applications with their work.

Although some measurement techniques have been developed during the rough rolling process, most of the related research summarized above has certain limitations due to harsh on-site conditions and the dynamic nature of slab processing. As a result, there are shortcomings in their practical application effectiveness. Therefore, it is of great significance to develop a real-time measurement system for measuring the head warping and lower buckling of rough-rolled slabs. This will lay the foundation for achieving intelligence in heavy industries.

Therefore, this paper proposes a fast and high-precision online detection system for detecting head warping and lower buckling of hot-rolled roughing slabs based on visual inspection. It provides specific values and defect types of head warping and lower buckling in real time for the production site, thereby providing adjustment basis for field operators to improve production efficiency and product quality. The system calculates the specific values of head warping and lower buckling of slabs based on the distance difference between slab corners and edge lines. Therefore, compared to other existing structured light visual inspection technologies, it has better real-time performance in practical applications. Industrial field application have proven that this system has high real-time performance, high detection accuracy, and high robustness, providing reliable reference for roughing plate shape control, thus improving product yield rate and rolling stability.

Currently, there is a lack of online real-time inspection for the quality of large-scale industrial products. The detection system described in this article mainly focuses on the dynamic real-time measurement of the appearance dimensions and defects of large-scale industrial products. The goal is to provide heavy-duty industrial equipment with eyes and brains. Therefore, this system has a wide range of application areas and prospects. 

## 2. Overall Architecture of the Online Detection System

**Figure 1:**
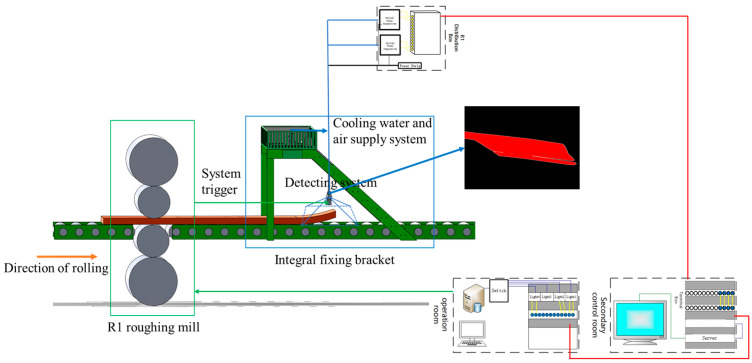
Overall architecture diagram of detection system.

The structure and installation schematic diagram of the online detection system for the head warping and lower buckling of rough-rolled slabs are shown in Figure 1. When the slab enters the roughing mill, the data from the mill system is transmitted to the inspection system. The CMOS line scan industrial camera is activated, and it captures images of the head and tail of the slab when it reaches the measuring position. Then, through a data transmission line, real-time image data is transferred to a server in the secondary control room over long distances. The server processes the images in real time, calculates specific types and values of head warping and lower buckling on the slab, and transmits this data to a large screen in the operation room for visualization. By using a mechanism-data dual-drive model, predictions can be made about future slabs’ bending and undercutting conditions, providing specific improvement solutions for onsite operators and allowing adjustments to be made to corresponding process parameters of roughing slabs. 

### 2.1. Optical Equipment

The optical equipment of this detection system mainly includes a CMOS array industrial camera, an industrial lens, an industrial switch, gigabit Ethernet cables, and single-mode optical fibers. The array industrial camera sensor adopts OnSemi PYTHON5000 (Beijing, China) with 5 million pixels and a resolution of 2592 × 2048. To meet the imaging requirements at long distances in industrial production sites, an 8 mm focal length industrial lens is selected. Due to the presence of alignment devices at both the entrance and exit of the roughing mill, there is a certain tilt angle when detecting roughing slabs in this described detection system.

### 2.2. The Detecting System for Fixed Devices

The detection system for fixed devices mainly include: a protective cooling camera fixing device, a universal joint, a fixed bracket, and a camera fixing board. Firstly, the industrial camera is fixed inside the protective cooling camera fixing device by a camera fixing board, and the protective cooling camera fixing device is fixed with the universal joint using screws. The universal joint is fixed to the fixed bracket with bolts, and the fixed bracket is bolted to the straightening machine platform from above. The bolts are reinforced by spot welding, thus achieving the fixation of the detection system in the production site. And it can be adjusted to any angle of the detection system through a universal joint.

## 3. Core Algorithm for Detecting Head Warping and Lower Buckling of Rough-Rolled Slabs

**Figure 2:**
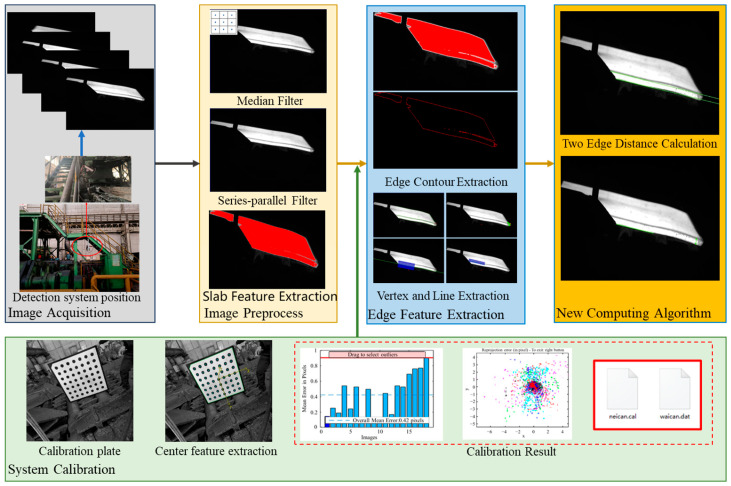
Algorithm flowchart for the detection system.

The algorithm flow of the online detection system for the head warping and lower buckling of hot-rolled rough slabs is shown in Figure 2. Firstly, the preparation work for detection is carried out by determining the intrinsic and extrinsic parameters of the industrial camera through calibration methods, generating calibration files, and determining the acquisition frequency and exposure level of the industrial camera based on the moving speed of rough-rolled slabs, network bandwidth, and production environment. When the slab enters the roughing mill, the secondary control system transmits signals to the detection system, triggering the CMOS array industrial camera in the detection system to start capturing images and performing real-time preprocessing on them. Then, using the Canny algorithm, edge detection is performed on the slab. Finally, precise extraction of vertex points and fitting of two edges of the slab are carried out to obtain accurate values for head warping and lower buckling by taking differences. This new method effectively reduces errors caused by changes in slab width or lateral position on moving rollers.

### 3.1. Measurement Principle

**Figure 3:**
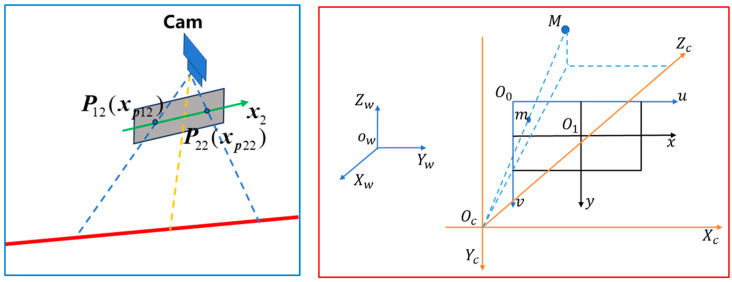
Detecting coordinate system relationships in the system.

The detection principles and methods of monocular vision have been extensively studied [20] and are relatively mature. The detection principle coordinate system is shown in Figure 3. $uO_{0}v$ is the image pixel coordinate system; $xO_{1}y$ is the image physical coordinate system, $O_{c}X_{c}Y_{c}Z_{c}$ is the camera coordinate system, and $O_{w}X_{w}Y_{w}Z_{w}$ is the world coordinate system.

The mathematical relationship between world coordinate system and camera coordinate system is as follows:

(1) $$\left[\begin{array}{c} X_{c}\\ Y_{c}\\ Z_{c}\\ 1 \end{array}\right]=\left[\begin{array}{cc} R & T\\ 0 & 1 \end{array}\right]\left[\begin{array}{c} X_{w}\\ Y_{w}\\ Z_{w}\\ 1 \end{array}\right]$$

The mathematical relationship between camera coordinate system and image physical coordinate system is as follows:

(2) $$s\left[\begin{array}{c} x\\ y\\ 1 \end{array}\right]=\left[\begin{array}{ccc} f & 0 & \begin{array}{cc} 0 & 0 \end{array}\\ 0 & f & \begin{array}{cc} 0 & 0 \end{array}\\ 0 & 0 & \begin{array}{cc} 1 & 0 \end{array} \end{array}\right]\left[\begin{array}{c} X_{c}\\ Y_{c}\\ Z_{c}\\ 1 \end{array}\right]$$

The mathematical relationship between the physical coordinate system of an image and the pixel coordinate system of an image is as follows:

(3) $$\left[\begin{array}{c} u\\ v\\ 1 \end{array}\right]=\left[\begin{array}{ccc} 1/dx & 0 & u_{0}\\ 0 & 1/dy & v_{0}\\ 0 & 0 & 1 \end{array}\right]\left[\begin{array}{c} X\\ Y\\ 1 \end{array}\right]$$

The mathematical relationship between the world coordinate system and the image pixel coordinate system is as follows:

(4) $$\begin{array}{ll} s\left[\begin{array}{c} u\\ v\\ 1 \end{array}\right] & =\left[\begin{array}{ccc} 1/dx & 0 & u_{0}\\ 0 & 1/dy & v_{0}\\ 0 & 0 & 1 \end{array}\right]\left[\begin{array}{ccc} f & 0 & \begin{array}{cc} 0 & 0 \end{array}\\ 0 & f & \begin{array}{cc} 0 & 0 \end{array}\\ 0 & 0 & \begin{array}{cc} 1 & 0 \end{array} \end{array}\right]\left[\begin{array}{cc} R & T\\ 0 & 1 \end{array}\right]\left[\begin{array}{c} X_{w}\\ Y_{w}\\ Z_{w}\\ 1 \end{array}\right]\\ & =\left[\begin{array}{ccc} f_{x} & 0 & \begin{array}{cc} u_{0} & 0 \end{array}\\ 0 & f_{y} & \begin{array}{cc} v_{0} & 0 \end{array}\\ 0 & 0 & \begin{array}{cc} 1 & 0 \end{array} \end{array}\right]\left[\begin{array}{cc} R & T\\ 0 & 1 \end{array}\right]\left[\begin{array}{c} X_{w}\\ Y_{w}\\ Z_{w}\\ 1 \end{array}\right] \end{array}$$

Due to the influence of the onsite environment, there is a slight tilt angle between the detection system and the cross-cutting board. In order to correct the skew error of the $uO_{0}v$ coordinate system’s axes, we introduce the formula $\gamma =f_{x}\cdot \tan\theta$, where θ indicates the degree of inclination of a plane along the v-axis. The Equation (4) can be written as:

(5) $$\begin{array}{ll} s\left[\begin{array}{c} u\\ v\\ 1 \end{array}\right] & =\left[\begin{array}{ccc} 1/dx & \gamma & u_{0}\\ 0 & 1/dy & v_{0}\\ 0 & 0 & 1 \end{array}\right]\left[\begin{array}{ccc} f & 0 & \begin{array}{cc} 0 & 0 \end{array}\\ 0 & f & \begin{array}{cc} 0 & 0 \end{array}\\ 0 & 0 & \begin{array}{cc} 1 & 0 \end{array} \end{array}\right]\left[\begin{array}{cc} R & T\\ 0 & 1 \end{array}\right]\left[\begin{array}{c} X_{w}\\ Y_{w}\\ Z_{w}\\ 1 \end{array}\right]\\ & =\left[\begin{array}{ccc} f_{x} & \gamma & \begin{array}{cc} u_{0} & 0 \end{array}\\ 0 & f_{y} & \begin{array}{cc} v_{0} & 0 \end{array}\\ 0 & 0 & \begin{array}{cc} 1 & 0 \end{array} \end{array}\right]\left[\begin{array}{cc} R & T\\ 0 & 1 \end{array}\right]\left[\begin{array}{c} X_{w}\\ Y_{w}\\ Z_{w}\\ 1 \end{array}\right] \end{array}$$

### 3.2. System Calibration Detection

**Figure 4:**
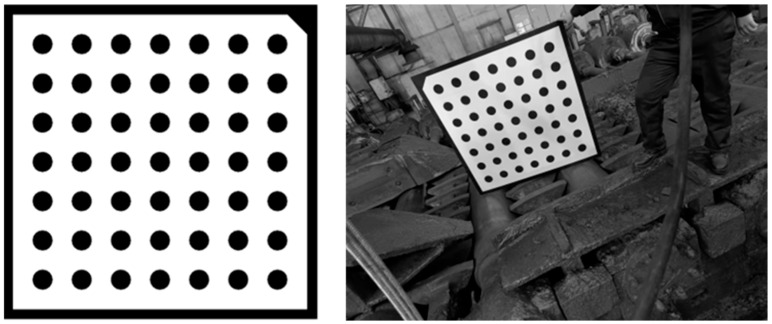
Design of calibration board.

On the basis of improving the detection principle, this paper adopts Zhang Zhengyou’s calibration algorithm [21] to calibrate industrial cameras. The calibration board adopts a 7 × 7 circular calibration board, and the size of the calibration board is 1000 × 1000 × 5 mm, as shown in Figure 4.

**Figure 5:**
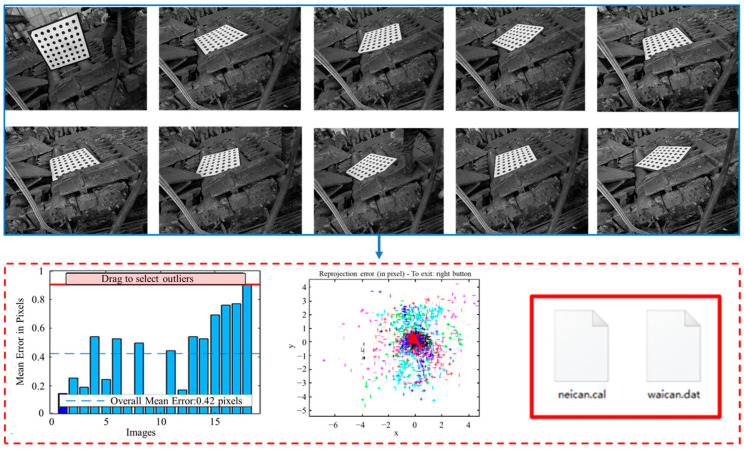
System calibration.

On-site calibration image acquisition involves the identification and processing of the circular centers in an image of a calibration board to establish the correlation between pixel coordinates and real-world coordinates. This enables the calculation of both intrinsic and extrinsic parameters for the camera, thereby completing the calibration process for the detection system, as shown in Figure 5.

### 3.3. Image Preprocessing

In the process of capturing images of rough-rolled slabs, image quality is influenced by numerous factors. In addition to hardware factors such as cameras, lenses, and lighting used for image acquisition, the harsh on-site environment also has a significant impact on slab images. Therefore, in visual measurement, image preprocessing is often an indispensable part that can greatly eliminate non-ideal information in the images and enhance their true features. Commonly used image preprocessing techniques include image filtering for noise reduction, threshold segmentation, and morphological processing [22].

#### 3.3.1. Median Filtering

**Figure 6:**
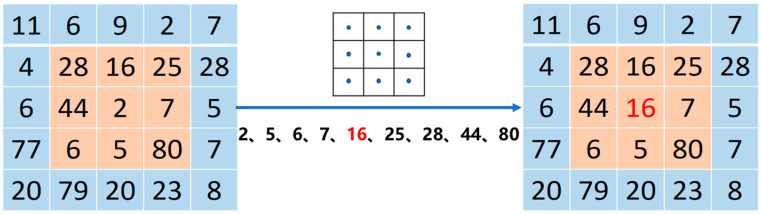
Principle diagram of median filtering.

The median filter is a method that overlays a certain shape of the median filtering window in a predetermined sequence on the original image, calculates the median value of all pixels in this region, and uses this value to represent the central pixel of the overlay area, as shown in Figure 6.

(6) $$G\left(x,y\right)=med\left\{f\left(x-k,y-l,k,l\in W\right)\right\}$$

**Figure 7:**
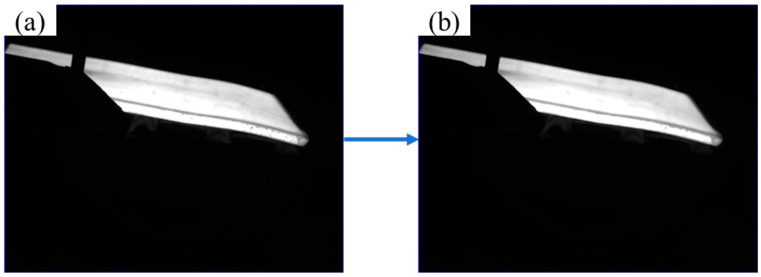
(**a**) master drawing; (**b**) median filtered image.

The filtering algorithm is simple and easy to implement, with minimal impact on the image boundaries, as shown in Figure 7.

#### 3.3.2. Feature Extraction of Rough Rolled Slabs

The extraction of image features is based on parameters such as grayscale and the shape, dividing the image into different sub-regions to remove unnecessary information and extract target features. The extracted image exhibits significant differences between different regions while demonstrating high similarity within the same feature. The automatic threshold segmentation algorithm is based on the grayscale histogram of an image to determine the gray threshold. It has a good feature extraction effect when there is not a significant difference in grayscale between the background and target [23]. This article adopts an adaptive threshold image feature extraction algorithm based on the maximum metric of inter-class variance.

Assuming that an image is automatically segmented into target feature A and the background with other interfering factors B, the pixel value distribution of the same category should be uniform, while the inter-class pixel value distribution should have significant differences [24]. Using variance to measure the uniformity or diversity of classes, an optimal threshold can maximize the variance between target class A and background class B [25].

The between-class variance $\sigma^{2}\left(K\right)$ can be represented as:

(7) $$\sigma^{2}\left(K\right)=P_{A}{\left(\mu_{A}-\mu \right)}^{2}+P_{B}\left(\mu_{B}-\mu \right)$$

In the equation: $\sigma^{2}\left(K\right)$ is the between-class variance;

K is the optimal threshold for maximizing variance;

$P_{A},\mu_{A},P_{B},\mu_{B}$ are the probabilities and means of the grayscale values appearing in two categories of pixels, A and B.

μ represents the average grayscale value of the overall image.

(8) $$\left\{\begin{array}{l} P_{A}=\sum_{i=1}^{K}P_{i}=P\left(K\right)\\ P_{B}=\sum_{i=K+1}^{K}P_{i}=1-P\left(K\right) \end{array}\right.$$

(9) $$\left\{\begin{array}{l} \mu_{A}=\frac{\sum_{i=1}^{K}iP_{i}}{P_{A}}=\frac{\mu \left(K\right)}{P\left(K\right)}\\ \mu_{B}=\frac{\sum_{i=K+1}^{M}iP_{i}}{P_{B}}=\frac{\mu -\mu \left(K\right)}{1-P\left(K\right)} \end{array}\right.$$

(10) $$\mu =\sum_{i=1}^{M}iP_{i}$$

**Figure 8:**
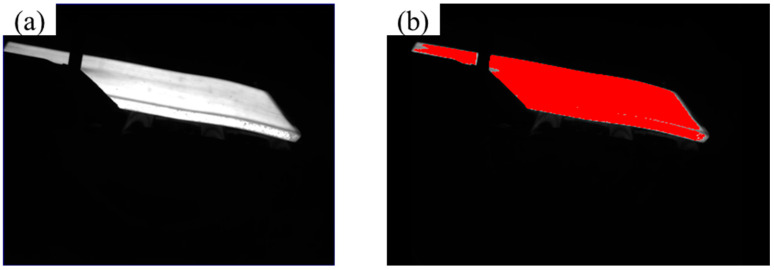
(**a**) master drawing; (**b**) feature extraction results of rough-rolled slabs.

The adaptive threshold image feature extraction algorithm can effectively distinguish the image target from the background, as shown in Figure 8.

#### 3.3.3. Novel Cascaded Filter Based on Morphological Processing

From Figure 8, it can be concluded that the extraction of cross-cutting board features has certain flaws due to environmental factors. These factors will greatly affect the subsequent high-precision measurement of cross-cutting board dimensions. In this paper, a novel cascaded filter is developed and constructed on the basis of morphological processing [26] to effectively remove interfering features other than cross-section board characteristics.

**Figure 9:**
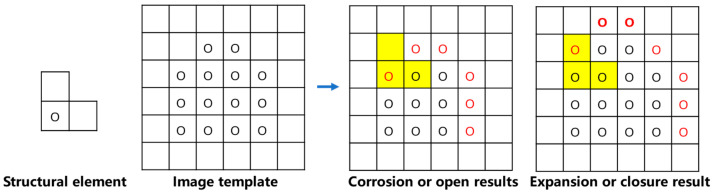
Morphological processing principles: yellow areas represent templates; red dots represent processing results.

As shown in Figure 9, basic methods of morphology processing is as follows: Image $f\left(x,y\right)$ performs erosion, dilation, opening operation, and closing operation on image $g\left(x,y\right)$.

Corrosion:

(11) $$\left(f\Theta g\right)\left(x\right)=\underset{y\in G}{\min}\left[f\left(x+y\right)-g\left(y\right)\right]$$

Inflation:

(12) $$\left(f⊕g\right)\left(x\right)=\underset{y\in G}{\max}\left[f\left(x-y\right)+g\left(y\right)\right]$$

Opening operation:

(13) $$\left(f\circ g\right)\left(x\right)=\left[\left(f\Theta g\right)⊕g\right]\left(x\right)$$

Closing operation:

(14) $$\left(f\cdot g\right)\left(x\right)=\left[\left(f⊕g\right)\Theta g\right]\left(x\right)$$

Cascaded morphological opening and closing filters: Open Close (OC), Close Open (CO).

Eliminate positive pulse signals:

(15) $$OC\left(f\left(x\right)\right)=\left(f\circ g\cdot g\right)\left(x\right)$$

(16) $$CO\left(f\left(x\right)\right)=\left(f\cdot g\circ g\right)\left(x\right)$$

**Figure 10:**
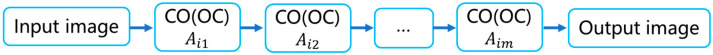
Cascade filter.

A new series filter suitable for cross-sectional board image processing was developed based on morphological open–close cascade filters. The principle of the filter is shown in Figure 10.

**Figure 11:**
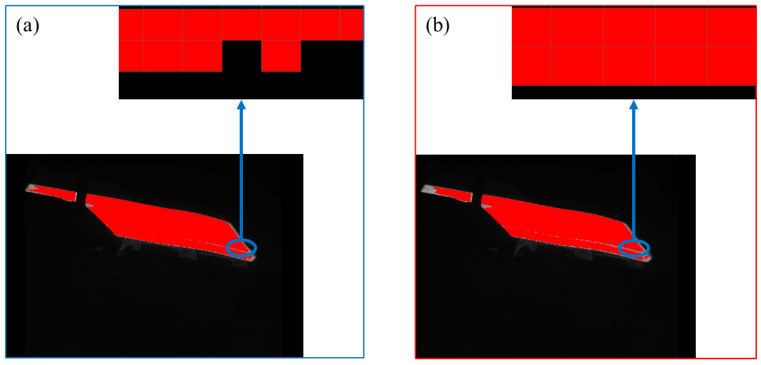
(**a**) slab features; (**b**) result of cascaded filter processing.

From Figure 10, it can be concluded that the novel cascaded filter based on morphology has a good effect in removing interference factors other than hot-rolled rough slab, and can accurately extract the features of hot-rolled rough slab, as shown in Figure 11.

#### 3.3.4. Comparison of Novel Cascaded Filter Based on Morphological Processing and Deep Learning

When processing the plate and strip images collected on the rough rolling site, it is necessary to first preprocess these images to ensure the accuracy and effectiveness of subsequent analysis. Specifically, we use manual labeling to carefully label each image and draw the contour line of the transverse strip. This process not only improves the recognizability of images, but also provides a necessary foundation for subsequent feature extraction based on deep learning.

**Figure 12:**
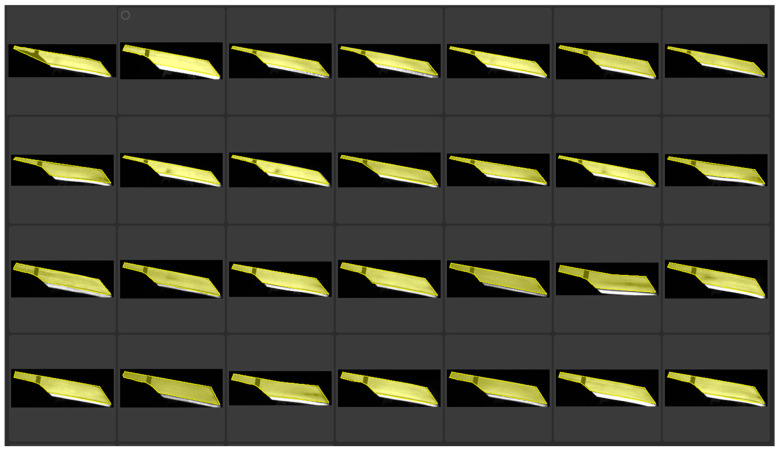
Model training.

After completing the manual labeling process, we divided the dataset into different parts, including the strip part and the background part, for the purpose of model training and evaluation. Specifically, 70% of the data will be used for training so that the model can learn sufficient features; 15% is used for validation to monitor the performance of the model and adjust hyperparameters; The remaining 15% will be used for testing, as shown in Figure 12.

**Figure 13:**
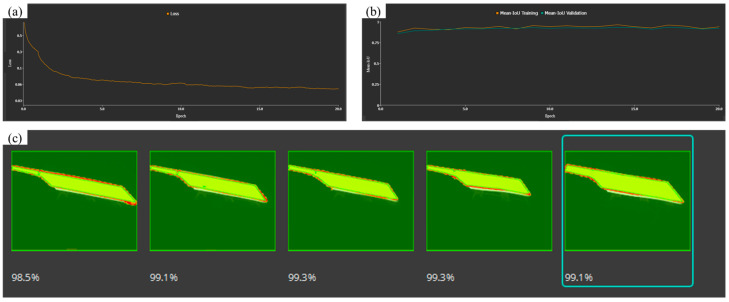
(**a**) model loss rate; (**b**) training and validation diagram; (**c**) identification result chart.

The model training results show that the loss rate is below 0.06, indicating that the performance of the model training results is relatively good and shows good convergence during the training process. A low loss rate symbolizes that the model can effectively learn the feature quantities in the dataset. However, in the deep learning process, the overfitting of data and errors in edge contour recognition, as shown in Figure 13.

**Figure 14:**
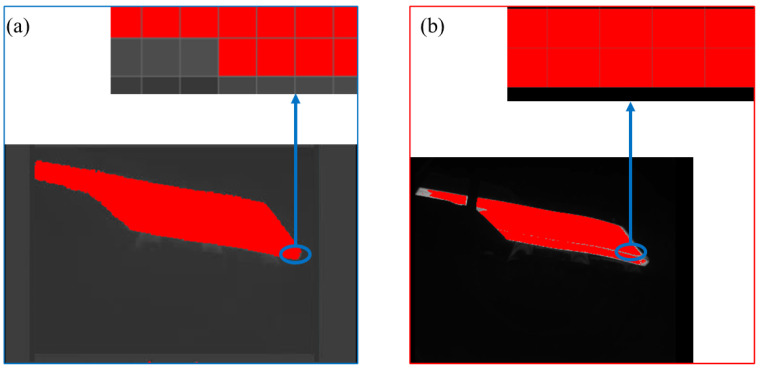
(**a**) result of deep learning; (**b**) result of cascaded filter processing.

As shown in Figure 14, the image feature map processed via deep learning is easier to be compared by the novel cascaded filter based on morphological processing. After using the novel cascaded filter based on morphological processing, the edge contour of the image slab is smoother. Therefore, the novel cascaded filter based on morphological processing is proved to be advanced and effective.

## 4. Methods for Measuring the Head Warping and Lower Buckling of Slabs

**Figure 15:**
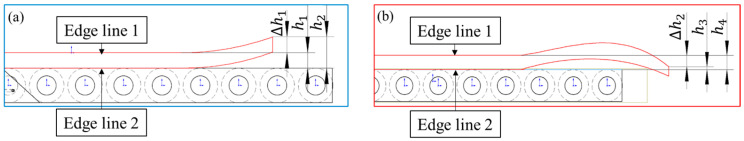
(**a**) calculation method of slab warping; (**b**) calculation method of slab lower buckle.

During the rough rolling process, if there is a situation where the slab has a hooked head, severe warping of the slab can cause it to collide with the working rolls, affecting their bite and causing steel stacking accidents. During transportation, the lower buckling of the slab constantly hits the roller conveyor, damaging it and creating a lot of noise. In severe cases, the head of the slab may even get stuck in the drainage ditch. Therefore, in order to achieve quantitative detection and classification of the head warping and lower buckling of the rough-rolled slab, it is necessary to obtain the difference between the distance from the vertex $P\left(x,y,z\right)$ on the head to edge line 1 and the distance to edge line 2. If the obtained value is positive, it indicates that the slab is warping; if it is negative, it indicates that the slab is lower buckling. As shown in Figure 15, $∆h_{1}$ represents the value of slab warpage, which is also the distance from point $P\left(x,y,z\right)$ to edge line 1; $h_{1}$ represents the distance between edge line 1 and edge line 2, which is also the thickness of the slab; $h_{2}$ represents the distance from point $P\left(x,y,z\right)$ to edge line 2; $∆h_{2}$ represents the value of slab lower buckling, $h_{3}$ represents the distance from point $P\left(x,y,z\right)$ to edge line 2; $h_{4}$ represents the distance between edge line 1 and edge line 2. The reason why the distance from point P to edge line 1 is not directly used as the value for measuring the warping and lower buckling of the slab in this article is because the width of the slab may vary, and there will inevitably be some errors in the detection system. However, by subtracting the distance from point $P\left(x,y,z\right)$ to edge line 2 from the distance between edge lines 1 and 2, we can effectively reduce these errors.

(17) $$\Delta h_{1}=h_{2}-h_{1}$$

(18) $$\Delta h_{2}=h_{3}-h_{4}$$

The distance $h_{2}$ from point $P\left(x,y,z\right)$ to the edge line 2. There are two points on the edge line, point 1 $M\left(x_{1},y_{1},z_{1}\right)$ and point 2 $M\left(x_{2},y_{2},z_{2}\right)$. First, I calculate the length and direction of the line. The direction is V=N−M, and the length is also $\left\|V\right\|$.

Then:

(19) $$h_{2}=\left\|(P-M)\times V\right\|/\left\|V\right\|$$

The distance $h_{1}$ between edge line 1 and edge line 2. For two points on edge line 1, point 1 $Q\left(x_{1},y_{1},z_{1}\right)$ and point 2 $R\left(x_{2},y_{2},z_{2}\right)$. First, I calculate the length and direction of the line. The direction is represented by V=R−Q and the length is represented by $\left\|V\right\|$. Any point on edge line 2 is denoted as W.

Then,

(20) $$h_{1}=\left\|(W-Q)\times V\right\|/\left\|V\right\|$$

Finally, we select the pair of points with the smallest distance.

(21) $$MinDistance=min\left(Distance1,\;Distance2\right)$$

Similarly, the solution methods for $∆h_{2},h_{3},h_{4}$ can be obtained.

### 4.1. Extraction of Rough-Rolled Slab Vertex and Edge Lines

**Figure 16:**
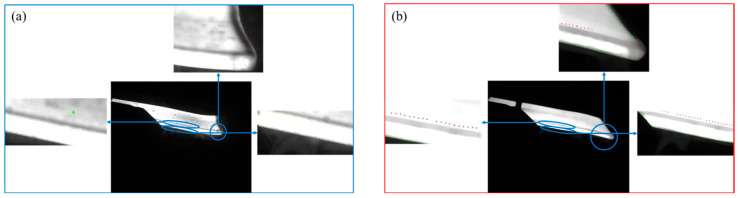
Extraction of edge contour points using the Canny algorithm: (**a**) warping; (**b**) buckle.

The parameters of the contour dimensions for roughing slab are based on the accurate extraction of the edge contour of the roughing slab, so it is particularly important to obtain accurate edge contours. Common edge detection algorithms include first-order Sobel operator, Roberts operator, second-order Laplacian operator, and the Canny operator [27]. Canny [28] algorithm provides more edge information in its detection results, with higher accuracy in locating extracted edges and stronger noise resistance. It produces rougher slab edges that are more complete, smooth, and of higher quality. Therefore, this paper chooses to use the Canny algorithm to extract the edge contours of roughing slabs. The main steps of the Canny algorithm [29,30,31] are as follows: Gaussian filtering to smooth the image → calculation of gradients and gradient directions → non-maximum suppression to retain points with maximum local gradient changes → dual thresholding for selecting edges. The extracted results of contour points are shown in Figure 16.

**Figure 17:**
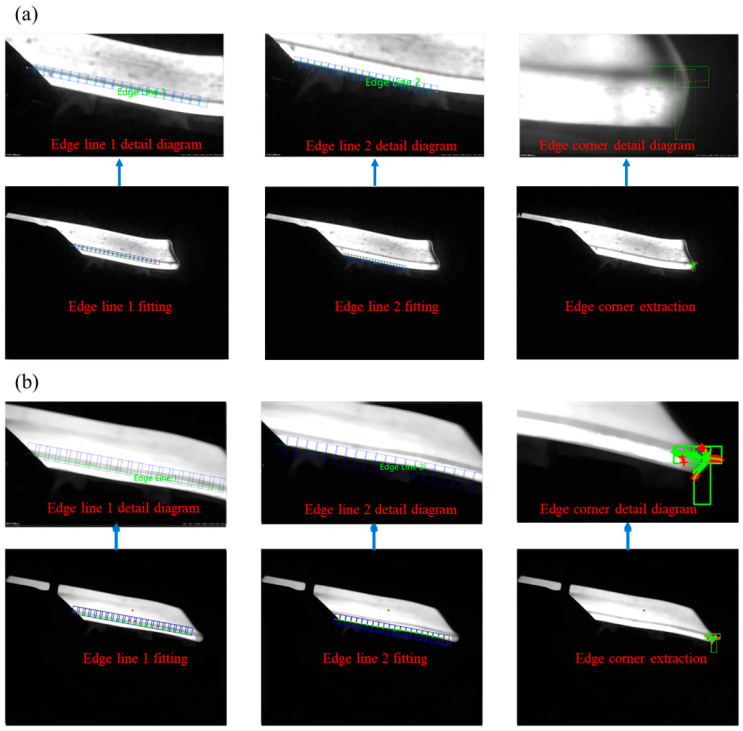
Extraction results of straight lines and vertex on the edge of the slab: (**a**) warping; (**b**) buckling.

The extraction results of the straight lines 1 and 2 at the edge of the slab, as well as the vertex *P*, are shown in Figure 17. The figure includes the extraction results of both warped and depressed edges of the slab. The figure includes the results of edge straightening and vertex for the warping and lower buckling of slab.

### 4.2. Calculation Results of Head Warping and Lower Buckling of Rough-Rolled Slabs

**Figure 18:**
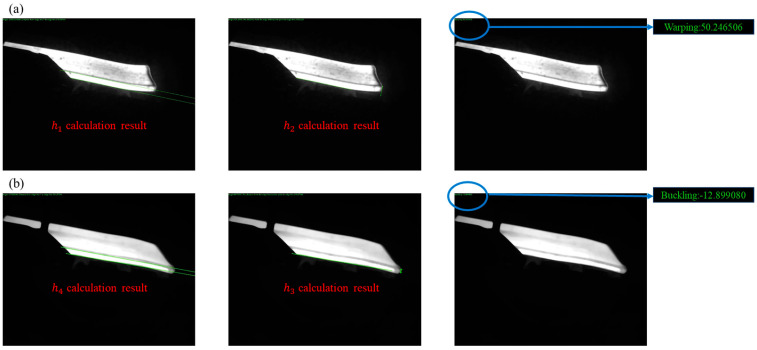
Calculation results of slab head warping and lower buckling: (**a**) warping; (**b**) buckling.

The accurate results of the slab’s edge straight line and the vertex of the head can be obtained through Equations (17)–(20), which can then be used to determine the precise curvature and undercutting of the slab, as shown in Figure 18.

From Figure 18, the detection results for the slab image case described in the article are summarized in Table 1. The positive and negative signs of the detection values represent different types of slab warping and lower buckling. Positive numbers indicate slab warping, while negative numbers indicate slab lower buckling. The industrial site requires a detection accuracy of ≤±5 mm. From the table below, it can be seen that the detection accuracies are 0.25 mm and 0.10 mm, which meet the requirements for industrial production.

## 5. Industrial Applications

**Figure 19:**
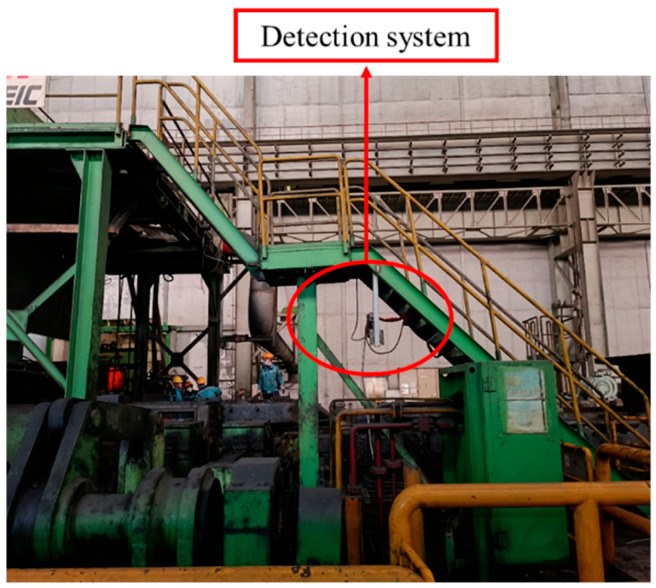
On-site equipment installation.

The detection system described in this article has been applied to industrial sites, mainly including on-site hardware installation, overall circuit arrangement, control system development, and software visualization interface, as shown in Figure 19. The thickness of different specifications of rough rolling slabs will be different, but their thickness difference is generally not >10 mm, the nominal thickness of the slab is 230 mm−240 mm. After three passes of R1 roughing mill rolling, the thickness of the slab will be 100 mm-150 mm. The detection range of the detection system described in this article is 20 mm-500 m, so any slab thickness in the field can be detected. The slab production speed is 2 m/s, and the maximum detection speed of the detection system is 5 m/s, so the production speed can be met.

### 5.1. Verify the Accuracy of the Detection System

To verify the detection accuracy of the inspection system for the head warping and lower buckling of slabs, in this paper, 50 images were collected for each of four kinds of rough-rolled slabs, and the actual values measured by manual field were compared with those measured by the detection system, and it was concluded that the detection system had high accuracy and stability. Here are the test results for four different steel grades:

#### 5.1.1. QSTE Series 1

In this paper, 50 rough-rolled slabs of QSTE series 1 are compared and analyzed by manual measurement and a detection system, and the results are shown in Table 2.

**Figure 20:**
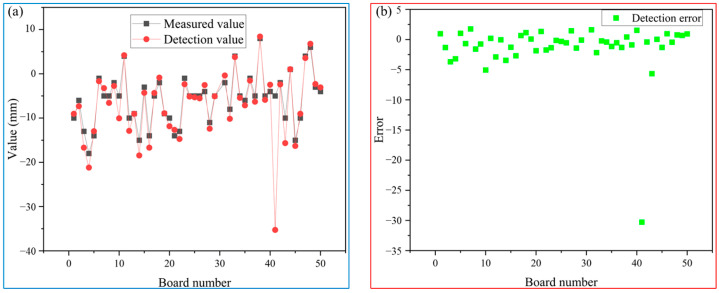
(**a**) comparison between detection values and measured values; (**b**) the error between detection values and measured values.

From Figure 20, it can be concluded that the detection accuracy of the measurement values and the detection system for 50 slabs is generally controlled within ±5 mm. Only one slab showed a significant deviation between the detected data and manual measurements, the reason is that the operator working in the field, mistakenly brought the high-temperature object into the field of view of the industrial camera, just with the slab being produced in the same image, so there is a large detection error of the slab. (From the side also reflects the sensitivity of the system and the ability to capture high-lighted objects). From Table 2, it can be observed that there was an incorrect judgment for one slab in terms of detection type compared to manual observation. This particular slab also had a large error in its detected value. However, all other slabs had consistent detection types with the actual situation.

#### 5.1.2. QSTE Series 2

In this paper, 50 rough-rolled slabs of QSTE series 2 are compared and analyzed by manual measurement and a detection system, and the results are shown in Table 3.

**Figure 21:**
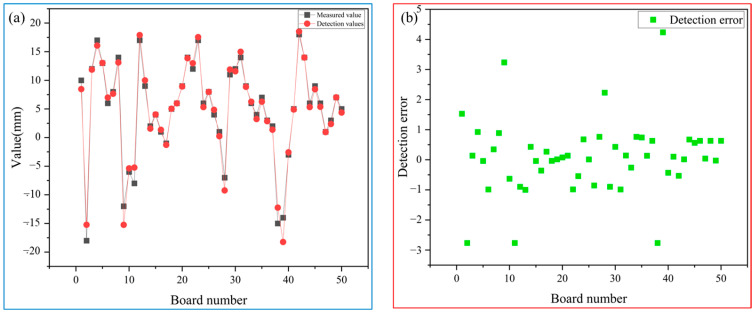
(**a**) comparison between detection values and measured values; (**b**) the error between detection values and measured values.

From Figure 21, it can be concluded that the detection accuracy of the measurement values and the detection system for 50 slabs is generally controlled within ±5 mm. From Table 3, in terms of detection type, the results obtained by the detection system are completely consistent with those obtained by manual detection.

#### 5.1.3. 700L

In this paper, 50 rough-rolled slabs of 700L are compared and analyzed by manual measurement and detection system, and the results are shown in Table 4.

**Figure 22:**
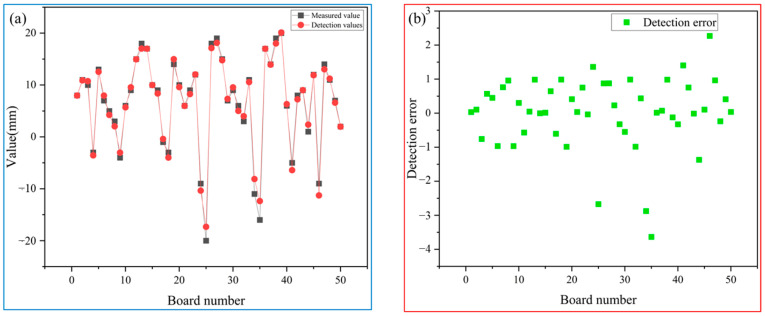
(**a**) comparison between detection values and measured values; (**b**) the error between detection values and measured values.

From Figure 22, it can be concluded that the detection accuracy of the measurement values and the detection system for 50 slabs is generally controlled within ±5 mm. From Table 4, in terms of detection type, the results obtained by the detection system are completely consistent with those obtained by manual detection.

#### 5.1.4. 510L

In this paper, 50 rough-rolled slabs of 510L are compared and analyzed by manual measurement and detection system, and the results are shown in Table 5.

**Figure 23:**
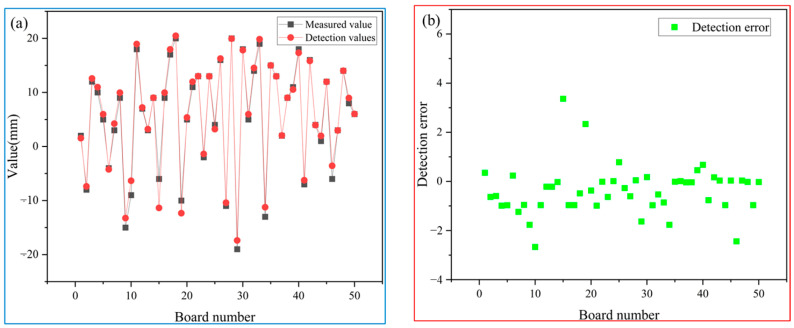
(**a**) comparison between detection values and measured values; (**b**) the error between detection values and measured values.

From Figure 23, it can be concluded that the detection accuracy of the measurement values and the detection system for 50 slabs is generally controlled within ±5 mm. From Table 5, in terms of detection type, the results obtained by the detection system are completely consistent with those obtained by manual detection.

According to the test results of four different steel grades, it can be concluded that the detection system has a good detection effect on the head warping and lower buckling of rough-rolled slab, and the detection error is less than or equal to 10 mm in the industrial field, so it can meet the needs of industrial field production.

### 5.2. Effectiveness and Expansion

At present, the system described in this paper has been successfully applied to the detection of rough rolling slabs in hot continuous rolling, which provides a fast and effective detection method for the rough rolling department of hot continuous rolling, provides data support for the control of field operators, greatly increases the production quality and efficiency of the slab, and also saves 1–2 manpower for the field rough rolling department. However, its application field can be more, such as: (1) the appearance of large thick plate contours, defects in online real-time detection, and detection accuracy to meet the needs of an industrial production site; (2) On-line real-time detection of the contour and the size of the outer wheel of the cross-cutting plate; (3) On-line real-time measurement of the geometric dimensions of a cold-rolled strip. For the detection of these large plates and strips, the detection accuracy of the system can meet their industrial requirements.

## 6. Conclusions

The article proposes a visual online detection system for the head warping and lower buckling of rough-rolled slab, which has been successfully applied in industrial field. The main content of this detection system is as follows:

(1) A detection system is constructed for industrial environments, where the testing system is set at a certain angle with the tested billet for detection. An applicable visual inspection coordinate transformation model has been established for industrial sites to effectively reduce detection errors caused by camera tilt.
(2) In response to the impact of environmental factors such as dust and iron filings in industrial sites, this article developed a cascaded filter based on morphological processing, which effectively removes noise from the field environment and smooth the edge profile of the slab, by comparing existing filtering techniques such as median filtering.
(3) Based on the implementation of the Canny algorithm for slab contour extraction, this article introduces a series of morphological filters to effectively remove redundant parts extracted by the Canny algorithm.
(4) The article proposes a method for calculating the head warping and lower buckling values of the slab, which determines the precise values by subtracting the distance from the corner point at the top of slab to the straight line at its lower edge from that between its upper and lower edges. This method can effectively reduce detection errors caused by changes in slab width and vibration.
(5) By application in industrial sites, the detection system has demonstrated excellent performance with stable operation and positive feedback from on-site operators. Furthermore, the measurement accuracy of the system has been verified to be ≤± 5 mm, while the type detection accuracy is ≥99%, meeting the precision requirements in industrial fields. Consequently, expansive research and application prospect is expected.

## Figures and Tables

**Table 1:** Results of head warping and lower buckling detection for rough-rolled slabs.

Board Number	Measured Value /mm	Detection Value/mm	Error/mm	Type
1	50	50.250000	0.25	Warping
2	13	−12.900000	0.10	Lower buckling

**Table 2:** Results of measured value and detection value.

Board Number	Measured Value	Measured Type	Detection Value	Detection Type
1	−10	Lower buckle	−9.035820	Lower buckle
2	−6	Lower buckle	−7.321800	Lower buckle
3	−13	Lower buckle	−16.684876	Lower buckle
4	−18	Lower buckle	−21.188221	Lower buckle
5	−14	Lower buckle	−12.959442	Lower buckle
6	−1	Lower buckle	−1.671528	Lower buckle
7	−5	Lower buckle	−3.234108	Lower buckle
……	……	……	……	……
49	−3	Lower buckle	−2.309645	Lower buckle
50	−4	Lower buckle	−3.068250	Lower buckle

**Table 3:** Results of measured value and detection value.

Board Number	Measured Value	Measured Type	Detection Value	Detection Type
1	2	Warping	1.536354	Warping
2	−8	Lower buckle	−7.369852	Lower buckle
3	12	Warping	12.594231	Warping
4	10	Warping	10.985465	Warping
5	5	Warping	5.968742	Warping
6	−4	Lower buckle	−4.236985	Lower buckle
7	2	Warping	1.536354	Warping
……	……	……	……	……
49	8	Warping	8.965741	Warping
50	6	Warping	6.023589	Warping

**Table 4:** Results of measured value and detection value.

Board Number	Measured Value	Measured Type	Detection Value	Detection Type
1	8	Warping	7.968547	Warping
2	11	Warping	10.896235	Warping
3	10	Warping	10.756581	Warping
4	−3	Lower buckle	−3.569726	Lower buckle
5	13	Warping	12.549876	Warping
6	7	Warping	7.965874	Warping
7	8	Warping	7.968547	Warping
……	……	……	……	……
49	7	Warping	6.589632	Warping
50	2	Warping	1.963546	Warping

**Table 5:** Results of measured value and detection value.

Board Number	Measured Value	Measured Type	Detection Value	Detection Type
1	2	Warping	1.536354	Warping
2	−8	Lower buckle	−7.369852	Lower buckle
3	12	Warping	12.594231	Warping
4	10	Warping	10.985465	Warping
5	5	Warping	5.968742	Warping
6	−4	Lower buckle	−4.236985	Lower buckle
7	2	Warping	1.536354	Warping
……	……	……	……	……
49	8	Warping	8.965741	Warping
50	6	Warping	6.023589	Warping

## Data Availability

Dataset available on request from the authors.
